# RAS mutations in myeloid malignancies: revisiting old questions with novel insights and therapeutic perspectives

**DOI:** 10.1038/s41408-024-01054-2

**Published:** 2024-04-24

**Authors:** Dana Alawieh, Leila Cysique-Foinlan, Christophe Willekens, Aline Renneville

**Affiliations:** 1 INSERM U1287, Gustave Roussy, Paris-Saclay University, Villejuif, France; 2 Department of Hematology, Gustave Roussy, Villejuif, France; 3 Department of Medical Biology and Pathology, Gustave Roussy, Villejuif, France

**Keywords:** Haematological cancer, Oncogenes

## Abstract

*NRAS* and *KRAS* activating point mutations are present in 10–30% of myeloid malignancies and are often associated with a proliferative phenotype. *RAS* mutations harbor allele-specific structural and biochemical properties depending on the hotspot mutation, contributing to variable biological consequences. Given their subclonal nature in most myeloid malignancies, their clonal architecture, and patterns of cooperativity with other driver genetic alterations may potentially have a direct, causal influence on the prognosis and treatment of myeloid malignancies. *RAS* mutations overall tend to be associated with poor clinical outcome in both chronic and acute myeloid malignancies. Several recent prognostic scoring systems have incorporated *RAS* mutational status. While *RAS* mutations do not always act as independent prognostic factors, they significantly influence disease progression and survival. However, their clinical significance depends on the type of mutation, disease context, and treatment administered. Recent evidence also indicates that *RAS* mutations drive resistance to targeted therapies, particularly FLT3, IDH1/2, or JAK2 inhibitors, as well as the venetoclax-azacitidine combination. The investigation of novel therapeutic strategies and combinations that target multiple axes within the RAS pathway, encompassing both upstream and downstream components, is an active field of research. The success of direct RAS inhibitors in patients with solid tumors has brought renewed optimism that this progress will be translated to patients with hematologic malignancies. In this review, we highlight key insights on *RAS* mutations across myeloid malignancies from the past decade, including their prevalence and distribution, cooperative genetic events, clonal architecture and dynamics, prognostic implications, and therapeutic targeting.

## Introduction

RAS proteins are a family of 21-kDa proteins that are at the heart of signaling pathways controlling various biological processes such as cell proliferation, differentiation, and survival. This family of proteins are specialized guanine nucleotide-binding and hydrolyzing molecules that belong to the small G-protein (GTP-ase) superfamily. They are encoded by highly related *RAS* genes, namely, *KRAS* (Kirsten rat sarcoma viral oncogene homolog), *NRAS* (neuroblastoma RAS viral oncogene homolog), and *HRAS* (Harvey rat sarcoma viral oncogene homolog), encoding 4 homologous proteins (sharing 85% sequence homology); H-RAS, K-RAS4A and K-RAS4B (two splice variants of K-RAS), and N-RAS [1]. Oncogenic mutations in RAS GTPases render the proteins constitutively GTP bound and active, promoting oncogenesis. However, the level of expression and activation of each specific RAS protein leads to different cellular responses and oncogenic phenotypes [2, 3]. Three well-studied RAS effectors are PI3-kinase (PI3K), Raf, and Ral-GDS proteins. Among these, the abnormal activation of the Raf/MEK/ERK pathway and the PI3K/Akt/mTOR cascade are strongly implicated in the development and maintenance of RAS-mutated cancers [4, 5].

*RAS* activating point mutations are found in nearly 20% of human cancers [5] and are highly prevalent in myeloid malignancies where they are often associated with a more proliferative phenotype [6, 7] and a more aggressive disease [8, 9]. While *RAS* mutation status has long been integrated into clinical decision making in patients with solid tumors, the clinical significance of *RAS* mutations in myeloid malignancies has only recently begun to be fully appreciated. Although considered as ‘undruggable’ in the past decade [10], significant progress in understanding RAS biology has brought us a step closer to identifying novel strategies for targeting *RAS*-mutated cancers, particularly in the context of myeloid malignancies.

This review aims to provide a detailed exploration of RAS mutations in myeloid malignancies including prevalent occurrences in acute myeloid leukemia (AML), myelodysplastic syndromes (MDS), chronic myelomonocytic leukemia (CMML), juvenile myelomonocytic leukemia (JMML), and myeloproliferative neoplasms (MPN). We address various challenges that have remained unanswered throughout the past decade. First, is how *RAS* mutations are not all equal; the type of the RAS mutated protein, the amino acid position, as well as the type of substitutions, varies across human cancers, including myeloid malignancies. Second, we decipher the cooperating genetic events with *RAS* mutations which modulate the resulting phenotype in mouse models. Third, we cover the clonal architecture and dynamics of *RAS* mutations. Lastly, we discuss variable prognostic implications depending on disease context, mutational type and the type of treatment administered. This highlights the challenge of RAS targeting therapies; due to their structural and biochemical properties, oncogenic RAS remains difficult targets for drug discovery.

## Prevalence and type of oncogenic mutations

**Fig. 1:**
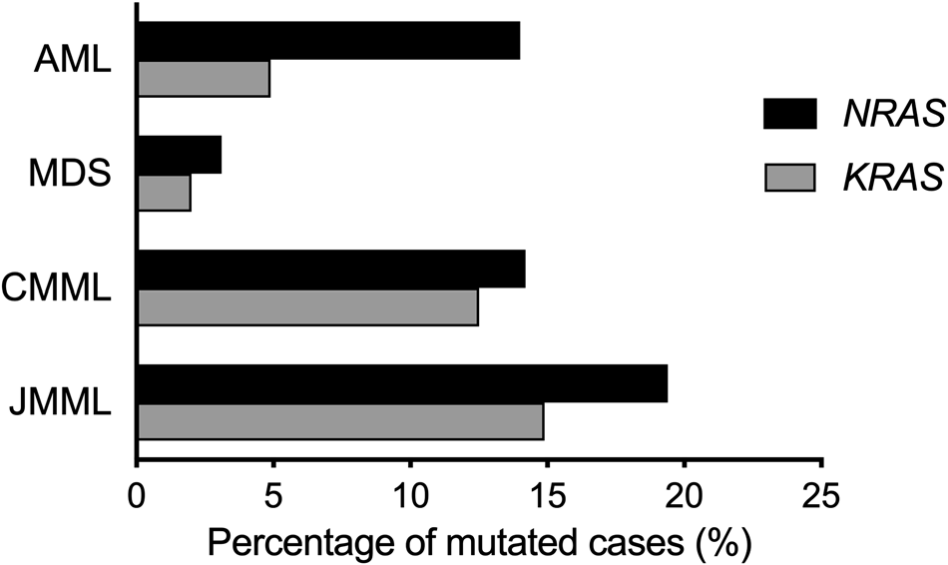
Prevalence of *NRAS* and *KRAS* mutations in myeloid malignancies. Percentage of mutated cases based on recent studies using high-throughput sequencing technologies and covering the entire *N/KRAS* coding sequence. AML: 1105 patients [12, 16]; MDS: 2957 patients [15]; CMML: 1540 patients (399 patients [15] and 1141 patients from unpublished personal data; JMML: 117 patients [13, 14, 17]. AML acute myeloid leukemia, MDS myelodysplastic syndromes, CMML chronic myelomonocytic leukemia, JMML juvenile myelomonocytic leukemia.

*RAS* mutations are prevalent in 10–30% of myeloid malignancies, with higher frequency in pediatric than adult diseases [11]. Among which, *NRAS* mutations are the most frequent, followed by *KRAS* mutations, whereas *HRAS* mutations are negligible in hematologic malignancies. The prevalence of *N/KRAS* mutations varies across different types of myeloid malignancies. According to recent studies using high-throughput sequencing technologies and covering the entire *N/KRAS* coding sequences (that is, exons 2, 3 and 4) [5, 12–17], MDS/MPN, notably CMML and JMML, harbor the highest incidence of *N/KRAS* mutations, ranging from 15% to 20% of cases for each gene. In adult MDS, both *N-* and *KRAS* mutations are identified in 2–3% of cases [15]. In AML, the overall prevalence of *RAS* mutations ranges between 15% and 20%. *RAS* mutations are particularly enriched in specific subsets of AML, such as AML with inv(3)/t(3;3) and AML with inv(16)/t(16;16), where these mutations are identified in around 30% and 35–40% of cases, respectively [15, 18–20]. In MPN, the prevalence of *RAS* mutations is very low in polycythemia vera (PV) and essential thrombocythemia (ET) (<1%), but can reach 6–8% in primary myelofibrosis (PMF) [21, 22] (Fig. 1).

**Fig. 2:**
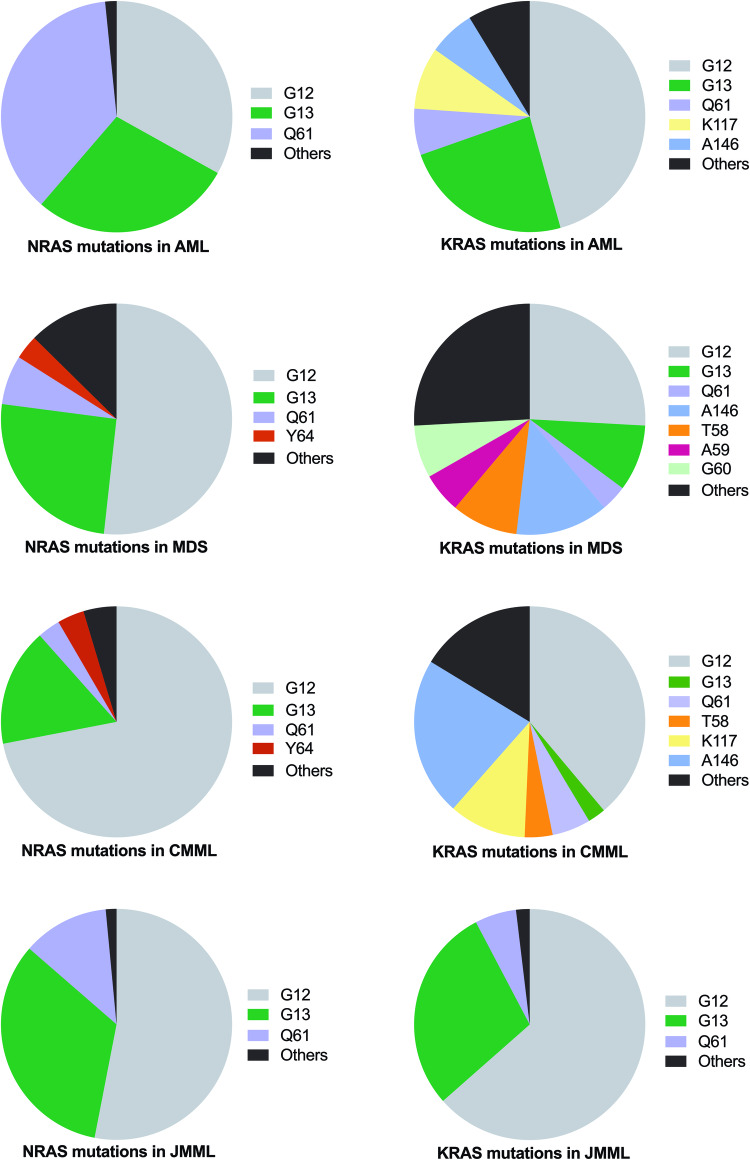
Distribution of *NRAS* and *KRAS* mutation type in myeloid malignancies. Distribution of the most frequently mutated codons in CMML (*n* = 1540), AML (*n* = 1105), JMML (*n* = 117) and MDS (*n* = 2975). The color code of each hotspot mutation is indicated on the right of each pie chart. The data are derived from the same patient cohorts as in Fig. 1.

Although more than 150 mutation sites have been reported in *RAS* genes, the most prevalent mutational hotspots are G12, G13, and Q61, accounting for approximately 80–95% of *NRAS* and 40–95% of *KRAS* mutations [5, 12–17]. *NRAS* and *KRAS* exhibit different hotspot preferences for G12, G13, Q61, and other non-canonical codons, such as T58, G60, K117, and A146, as illustrated in Fig. 2. Recent experimental evidence supports the notion that *RAS* mutations harbor allele-specific structural and biochemical properties, contributing to variability in biological consequences [23, 24].

*RAS* mutation patterns vary across different types of myeloid malignancies and even across disease subtypes. In AML, *NRAS* mutations equally affect G12, G13, and Q61 codons, each of the 3 amino acids representing about one third of all mutations, while *KRAS* mutation distribution displays less Q61 mutations and more rare variants, such as those involving K117 and Q146 codons. In core binding factor (CBF) AML particularly in AML with inv(16)/t(16;16), codon Q61 is much more frequently mutated than other codons. In contrast, *NPM1* mutations preferentially associate with *NRAS* G12/13 but not with *NRAS* Q61 mutations. In adult chronic myeloid disorders, including CMML and MDS, *NRAS* mutations are predominantly found at G12 codon, accounting for 50–70% of cases, while *KRAS* mutations show much more diversity in terms of amino acid positions. In JMML, more than 80% of *N/KRAS* mutations affect G12 and G13 codons (Fig. 2). Altogether, *RAS* mutation patterns in myeloid malignancies are likely shaped by quantitative and qualitative differences in the activation of downstream signaling pathways, as suggested in the “sweet spot” model proposed by Li et al. [3]. Although the underlying biological mechanisms of codon-preferential *RAS* mutations in specific types of myeloid malignancies remain poorly understood, it is likely that distinct mutagenesis and/or selection processes are involved in different clinical settings. Properly identifying and understanding the roles and nuances of different *RAS* mutations could potentially guide targeted therapies and ultimately improve patient outcomes.

## Modeling Ras mutations and their cooperation with other gene mutations

*Ras* mouse models have been extensively employed in hematologic malignancies with the aim of conducting in vivo experiments for disease understanding and preclinical trials. Table 1 provides an updated compilation of the recent Ras mouse models and their respective phenotype. Distinct *Ras* mutations exhibit variable behavior, for instance, the induction of heterozygous *Kras*^G12D/+^ expression in the hematopoietic system alone through Mx1-Cre leads to a rapid and highly penetrant myeloproliferative disease (MPD) modeling human MDS/MPN, but does not lead to AML progression [25]. In parallel, induction of the heterozygous *Kras*^A146T/+^ mutation in the hematopoietic compartment also led to an MDS/MPN phenotype similar to *Kras*^G12D^ mice, but with a significantly delayed onset [23]. In contrast, endogenous heterozygous *Nras*^G12D^ expression exhibits a modest and variable myeloid phenotype, although mice that are homozygous for a conditional *Nras*^G12D^ knock-in allele model aggressive MPN [26]. When expressed in the hematopoietic compartment, *Nras*^G12D^ alone induces a MPD similar to *Kras*^G12D^ but with significantly longer disease latency and lower penetrance [27–30]. These findings collectively suggest that discrepancies in the mutation type, and/or expression levels of distinct Ras proteins influence the severity of myeloid growth dysregulation [4]. The cooperation of Ras mutations with other genetic alterations, such as *Tet2*, *Dnmt3a*, or *Tp53* mutations, has also been recently investigated in mouse models (Table 1). Complete *Dnmt3a* loss enhances self-renewal in hematopoietic stem cells (HSCs), impairs differentiation, but is not sufficient to drive leukemogenesis in mice; specific disease progression depends on additional genetic alterations, such as *Ras* mutations [31, 32] Thus, complete loss of *Dnmt3a* synergizes with *Kras*^G12D^, expediting disease progression and culminating in approximately 30% of mice developing AML [30] (Table 1). Concurrently, *Nras*^G12D^, in conjunction with heterozygous *Dnmt3a* loss, promotes AML onset in one-third of the induced mice, providing a potentially more biologically pertinent representation given the prevalent heterozygosity of *DNMT3A* mutations in human disease. Alternatively, hotspot *Dnmt3a*^R878H^ mutation with *Nras*^G12D^ led to a much earlier onset in mice, shorter lifespan, and more severe AML-like disease [33]. This suggests that the type of *DNMT3A* mutation, along with acquisition of *RAS* mutations, could significantly promote the leukemogenic transformation and proliferation of HSCs.

**Table 1:** Mouse models of myeloid malignancies characterized by Kras or Nras mutations, with or without a cooperative genetic alteration in *p53*, *Dnmt3a*, *Tet2*, *Bcor*, or *Cux1*.

*Ras* gene	Amino acid modification	Cooperative abnormality	Mouse model type	Main disease characteristics	Pathological relevance	References
*Kras*	G12D	–	Het. Cond. KI Mx1-Cre; Kras^G12D/+^	Anemia, leukocytosis, splenomegaly, and myeloid hyperplasia in BM	Aggressive MPN	[25, 29]
*Kras*	A146T	–	Het. Cond. KI Mx1-Cre; Kras^A146T/+^	Anemia, mild splenomegaly, dysplastic morphology	MDS/MPN	[23]
*Nras*	G12D	–	Het. Cond. KI Mx1-Cre; Nras^G12D/+^	Anemia, splenomegaly, monocytosis	CMML, MPN	[26, 27]
*Nras*	G12D	–	Hom. Cond. KI Mx1-Cre; Nras^G12D/G12D^	Peripheral leukocytosis, splenomegaly, mature myeloid cells infiltration in hematopoeitic and non hematopoeitic tissue	Aggressive MPN	[22, 23]
*Kras*	G12D	*Dnmt3a*^*−/−*^	Het. Cond. KI Mx1-Cre; Kras^G12D/+^ Hom. Cond. KO Dnmt3a^fl/fl^ -Mx1-cre	Splenomegaly, myeloblast accumulation, monocyte/neutrophil expansion, defective erythroid and megakaryocyte development	MPN, AML	[31, 32]
*Nras*	G12D	*Dnmt3a*^R878H/+^	Het. Cond. KI Mx1-Cre; Nras^G12D^ Het. Cond. KI Mx1-Cre; Dnmt3a ^R878H/+^	Anemia, splenomegaly, leukocytosis, myelomonocytic, morphologically myeloblastic, increased circulating blasts and organ infiltration	AML	[33]
*Nras*	G12D	*Tet2*^*−/−*^	Het. Cond. KI Mx1-Cre; Nras^G12D/+^ Het. Cond. KI Mx1-Cre+;Tet2^fl/fl^	Leukocytosis, splenomegaly, monocytosis, thrombocytopenia, decreased erythroid compartment	CMML	[34]
*Nras*	G12D	*p53*^−/−^	Het. Cond. KI Mx1-Cre NrasLSL^G12D/+^ p53^fl/fl^ Mx1-Cre	Anemia, monocytosis, thrombocytopenia, immature myeloid compartment expansion in spleen, hepatosplenomegaly	AML and T-ALL	[36]
*Nras*	G12D	*p53*^R172H/+^	Het. Cond. KI Mx1-Cre NrasLSL-^G12D/+^ p53LSL-^R172H/+^	Splenomegaly, imbalanced myelopoiesis and lymphopoiesis, accumulation of myeloid blast cells in spleen and liver	AML	[36]
*Kras*	G12D	*Bcor* ^ΔE9−10^	Bcor fl/fl, flanked exons 9 and 10 leading to protein inactivation	Leukocytosis, splenomegaly and increased leukemic blasts in the PB and BM	AML	[37]
*Nras*	G12D	*Cux1*^low^	shRNA-based Cux1-knockdown mouse lines: Cux1^low^ (42% residual Cux1 mRNA in LSK cells)	Anemia, leukocytosis, morphologically myeloblastic or monocytic, increased circulating blasts and organ infiltration	MDS/MPN similar to JMML/CMML, developping high risk AML	[38, 39]

*Het. Cond. KI* Heterozygous conditional Knock-In, *Het. Cond. KO* Heterozygous conditional Knock-Out, *Hom. Cond. KI* Homozygous conditional Knock-In, *LSK* Lin^−^ Sca1^+^ c-Kit^+^, *BM* bone marrow, *PB* peripheral blood, *MPN* myeloproliferative neoplasm, *MDS* myelodysplastic syndrome, *CMML* chronic myelomonocytic leukemia, *AML* acute myeloid leukemia, *T-ALL* T-cell acute lymphoblastic leukemia, *JMML* juvenile myelomonocytic leukemia.

*Tet2*^*−*/−^ and *Nras*^G12D^ in hematopoietic cells synergize in vivo, engendering a lethal CMML-like disease with elevated self-renewal potential compared to mice harboring either mutation alone. Upon acquisition of the *Nras* mutation, clonal expansion is observed, precipitating leukemia progression and heightened sensitivity to GM-CSF [34]. These findings were validated in the context of *Tet2* haploinsufficiency and *Ras* mutations where they collaborate to disrupt hematopoietic stem and progenitor cells (HSPCs), inducing a lethal and significantly penetrant CMML-like disorder. The concurrent *Nras* and *Tet2* mutations also evoke cytokine hypersensitivity in HSPCs [35].

In the context of *Nras*^G12D/+^ associated with *p53* mutations, *Nras*^G12D/+^ x *p53*^−/−^ mice developed mixed AML and T-cell malignancy, whereas *Nras*^G12D/+^; *p53*^R172H/+^ mice rapidly developed a lethal AML with full penetrance and a median survival of ~80 days. Additionally, *Nras*^G12D/+^; *p53*^R172H/+^ HSPCs show imbalanced myelopoiesis and lymphopoiesis. It has also been reported that mutant *p53* and oncogenic *Nras* cooperatively dysregulate hematopoietic transcription factor networks and promote inflammation via NfkB [36]. This demonstrates that *Nras* mutations cooperate with *p53* mutants to promote AML in a much more important manner than either mutation alone.

*BCOR* mutations have been identified in various hematologic malignancies including MDS and AML. *Bcor* inactivation in aged mice was not sufficient for leukemogenesis but was associated with a significant increase in the absolute number of bone marrow myeloid progenitors. In contrast, *Bcor*^KO^ mice in cooperation with *Kras*^G12D/+^ developed a leukemia-like phenotype (Table 1). Additionally, the survival of *Bcor*^*KO*^;*Kras*^G12D/+^ mice was significantly reduced compared with *Kras*^G12D/+^ controls indicating that *Bcor* inactivation resulted in functional co-operation with oncogenic *Kras* to initiate leukemia in vivo [37].

*CUX1* mutations are common in myeloid neoplasms and significantly co-occur with oncogenic mutations in *RAS, PTPN11*, or *CBL* [38]. In murine models, *Cux1* deficiency gives rise to MDS-like phenotype but falls short of driving AML independently. However, mice bearing *Nras*^G12D^ and *Cux1* knockdown concurrently exhibited AML development, an outcome absent in mice with either mutation alone. The oncogenic influence of *Ras* drives an increase in self-renewal in *Cux1*-deficient HSPCs. Conversely, *Cux1* knockdown intensifies Ras signaling by mitigating negative regulators of RAS/PI3K signaling. Table 1 describes the phenotype of the resulting *Cux1*^*low*^*;Nras*^*G12D*^ mice, which mimic an AML-like disease compared to *Cux1*^*mid*^*;Nras*^*G12D*^ mice which are more MDS/MPN similar to that of JMML/CMML, indicating that the further decrease of *Cux1* expression drives a more penetrating phenotype in cooperation with *Nras*^G12D^ to drive AML [39]. Of note, all double mutant mice in Table 1 have significantly reduced survival as compared to mice harboring each mutation alone.

Taken together, murine models in myeloid malignancies have resulted in highly significant advancements in understanding how *Ras* mutations serve as cooperating mutations with other disease-initiating mutations. While *Ras* mutations alone do contribute to a significant myeloproliferative phenotype, they require cooperation with other mutations, more particularly those of tumor suppressor genes to drive leukemogenesis. The above examples underscore that the specific type of the cooperating mutation, in conjunction with *Ras* mutations, can yield diverse pathologic outcomes.

## Clonal architecture and evolution

The consequence of the type and order of mutation acquired leads to the HSC being more or less likely to facilitate subsequent acquisition of mutations and leukemia development. Recent research prompted inquiry into how such clones facilitate the acquisition of other mutations in signaling pathways, such as RAS, to enhance their clonal fitness [40, 41]. In the context of age-associated myeloid malignancies, *RAS* mutations tend to emerge exclusively in the context of other clonal hematopoiesis mutations suggesting that these late events may cooperate with founder mutations to drive the progression of clonal hematopoiesis toward malignancy, aligning with a stepwise model of leukemogenesis [40, 42].

In clonal hematopoiesis of indeterminate potential (CHIP), *RAS* mutations only occur secondarily in the presence of other mutations strongly correlated with the apparition of a hematologic malignancy. Unlike most age-associated MDS/MPN where *RAS* mutations are often observed to be subclonal, JMML is essentially a RASopathy arising through the acquisition of de novo signaling mutations or in the context of germline predisposition syndromes. Recent evidence suggests that very few somatic events are required for JMML leukemogenesis and confirmed the predominant role of RAS pathway alterations in disease initiation. RAS-activating mutations might have distinct effects on epigenome remodeling possibly correlated with disease aggressiveness [42–45]. CMML however, is a RASopathy of the elderly often found in a background of epigenetic and splicing alterations. In the context of AML, *N/KRAS* mutations may function as an early/initiating event but mostly as cooperating mutations acquired during disease progression. Regarding MDS and MPN, *RAS* mutations mainly appear as a late event, driving progression and transformation to AML.

Various studies have attempted to replicate the sequential addition of *Ras* mutations within different mutational contexts. For instance, HSCs acquiring *Runx1::Runx1T1* gain a competitive advantage, which leads to an expansion in the number of HSCs, thereby increasing the pool of cells capable of acquiring additional mutations like *Kras*. Ultimately, this promotes the development of leukemia and mimics the disease phenotype in mice. Conversely, HSCs that only acquire a *Kras* mutation, whether alone or in combination with *Runx1::Runx1T1*, are depleted due to loss of quiescence and self-renewal. This observation may elucidate why signaling mutations like *RAS* are not typically detected in pre-leukemic HSCs in AML patients; they tend to manifest as a later event in the leukemogenesis process. *Ras* mutations may necessitate cooperation with other mutations to confer this particular phenotype and are insufficient to do so as a solitary mutation [46]. This leads to the conclusion that the timing of emergence of *Ras* mutations in the clonal evolution is vital for the cell’s fate to transformation. However, earlier studies argue that *Ras* mutations alone partially enhance competitiveness of the HSC and promote pre-leukemic clonal expansion. It has been reported that *Nras*^G12D/+^ has a bimodal effect on HSCs in mice, increasing self-renewal potential and reducing division in one HSC subset while increasing division and reducing self-renewal in another HSC subset. Short-lived but rapidly dividing *Nras*^G12D/+^ HSCs presumably outcompete wild-type HSCs and are replenished over time by quiescent *Nras*^G12D/+^ HSCs [47]. Given that heterogeneity within HSCs is likely governed by various mechanisms of gene expression control, epigenetics and RNA splicing, variations in methylation levels and patterns give rise to stochastic transcriptional heterogeneity among genetically identical cells which may or may not protect the cell from external stress and the potential of acquiring further mutations. This heterogeneity could also elucidate the differing outcomes observed when HSCs are transformed by the same oncogenic event such as *N/KRAS* mutations. This suggests that the expansion of an *NRAS* mutant clone may be contingent on a specific cellular state [48], or possibly a chromatin state depending on which epigenetic factors are mutated [40].

Dormancy may be another factor influencing the emergence of RAS subclones. Dormant HSCs are normally in a quiescent state and are resistant to acute stress, but chronic stress such as infections, metabolic stress, or cytokine-related inflammation can exhaust them. Leukemic HSC are reported to co-opt physiological mechanisms of HSC sustenance to overcome this exhaustion, dominating normal HSCs in the niche and rendering them more fit. Moreover, mutant HSCs such as *Tet2*^−/−^ or *Dnmt3a*^−/−^ are also reported to secrete IL1-β and IFN-γ, allowing mutated clones to outcompete non-mutated clones. The niche thus becomes predominantly mutated, giving rise to both dormant, and active HSCs, which are more susceptible to proliferative signals [48]. Taken together, this raises the following hypotheses: does the exposure of mutant HSCs to chronic stress lead to epigenetic modifications rendering the clone more susceptible to acquire a *RAS* mutation? Does the harsh inflammatory *milieu* lead to a selection pressure of the *RAS*-mutated clone to evolve and expand? Could this explain why in JMML, a single initiating driver event is sufficient to drive leukemogenesis? Perhaps the cellular state, and cytokine milieu in utero is favorable for the competitive phenotype of the mutation, and the environment was predisposed to an infection or inflammation which rendered the clone to expand.

The literature currently presents a contradictory perspective regarding whether *RAS* mutations can induce clonal expansion independently or if they require a predisposed mutational context to manifest. Nevertheless, the arrangement of clonal populations may potentially have a direct, causal influence on the prognosis and treatment of myeloid malignancies [49]. Conversely, it is plausible that clonal architecture and the microenvironment might serve as a surrogate for an underlying process that itself contributes to chemoresistance or relapse. This highlights the need for more comprehensive research of the time-dependent consequences of *Ras* mutation emergence in the clonal architecture of leukemogenesis. Novel knock-in models facilitating the sequential introduction of mutations hold significant promise for future advancements. Additionally, deciphering the unique molecular signatures linked to pre-leukemic mutations in HSCs could pave the way for potential therapeutic advances aimed at selectively targeting the expansion of preleukemic stem cells. Exploring evolutionary dynamics through single-cell technologies and mathematical modeling holds the potential to enhance our comprehension of leukemic transformation and treatment resistance. This approach may also pave the way for the development of innovative therapeutic strategies and the identification of valuable biomarkers.

## Prognostic implications

*N/KRAS* mutations are significant contributors to the pathogenesis, progression, and often prognosis of myeloid malignancies. They are quite infrequent in the context of CHIP, with relatively low variant allele frequencies at 1% and 2%, respectively. The late emergence of a *RAS*-mutated clone however, is associated with a 12-fold elevated risk of developing a myeloid malignancy (Table 2). While further research is needed to strengthen this finding, it implies that individuals harboring a *RAS*-mutated CHIP clone require careful clinical monitoring due to their high-risk profile [40].

**Table 2:** Prognostic implications of RAS mutations in clonal hematopoiesis and myeloid malignancies.

Condition	Gene mutation	Prognostic score	Risk of relapse	Treatment response	Disease progression	Prediction of acute transformation	Prognostic impact on OS	References
Adult malignancies								
CHIP	*KRAS*	NA	NA	NA	Yes: HR = 12.4 (2.9–52.4)		No	[40]
	*NRAS*							
MDS	*KRAS*	IPSS-M	No	No significant difference with anthracyclines, conflicting results with HMA based treatment	Yes: HR = 1.42 (1.05–1.93)	No: HR = 1.22 (0.84–1.77)	Yes: mOS 16 vs 92 months (*P* *<* 0.01)	[8]
	*NRAS*				Yes: HR = 1.52 (1.05–2.20)	Yes: HR = 1.93 (1.25–2.98)		[15]
CMML	*KRAS*	No	No	No	Yes	No	No	[57]
	*NRAS*	CPSS-Mol	Yes: higher risk after HMA therapy (*P* = 0.003)	Yes: shorter OS and NRM after allo-SCT HR = 1.63 (1.15–2.31)		Yes: OR = 2.7 (1.4–5.3)	Yes: HR = 2.19	[58]
		CMML transplant score						[59]
PMF	*KRAS*	No	NA	Yes: lower responses on symptoms and spleen at 6 months after JAKi	Yes	Yes: 5-year CuI of LT 29,1% vs 8.8% (*P* < 0.0001)	Yes: mOS 55 vs 110 months (*P* = 0.01)	[21]
	*NRAS*							[22]
AML	*KRAS*	No	Yes: higher risk of relapse after HMA and HMA + VEN	Yes: higher risk of refractory disease with HMA and HMA + VEN	NA		Yes: only with non-intensive regimen, mOS 12 vs 30 months (*P* < 0.001)	[66]
								[65]
								[69]
	*NRAS*							[68]
Pediatric malignancies								
JMML	*KRAS*	NA	Yes: low risk of relapse after allo-SCT	Yes: lower relapse risk after allo-SCT and good response to low-dose AZA	Yes: rate of progression dependent on mutational status	NA	No	[11]
	*NRAS*		Yes: high relapse rate after allo-SCT	Yes: high relapse rate after allo-SCT				[43]
AML	*KRAS*	NA	Yes: mEFS 5.6 vs 22.8 months		NA	NA	Yes: mOS 22.5 vs 124 months	[72]
	*NRAS*							

*CHIP* clonal hematopoiesis of indeterminate potential, *MDS* myelodysplastic syndrome, *CMML* chronic myelomonocytic leukemia, *PMF* primary myelofibrosis, *AML* acute myeloid leukemia, *JMML* juvenile myelomonocytic leukemia, *NA* not available, *OS* overall survival, *mOS* median OS, *EFS* event-free survival, *mEFS* median EFS, *NRM* non-relapse mortality, *HMA* hypomethylating agent, *Ven* venetoclax, *JAKi* JAK inhibitor, *allo-SCT* allogeneic stem cell transplantation, *HR* hazard ratio, *OR* odd ratio, *5-year CuI of LT* five year-cumulative incidence of leukemic transformation survival, *IPSS-M* Molecular International Prognostic Scoring System, *CPSS-Mol* clinical-molecular CMML-specific prognostic scoring system.

In MDS, *RAS* mutations are correlated with more aggressive disease subtypes, higher IPSS-M risk, and reduced event-free survival (EFS) and overall survival (OS). *RAS*-mutated MDS patients are also reported to have an OS of only 16 months *versus* 92 months in non-RAS-mutated MDS patients [8]. This has been validated in a separate cohort where patients have an increased risk of leukemic transformation, primarily associated with *NRAS* rather than *KRAS* mutations [15] (Table 2). One hypothesis is that the rarer occurrence of *KRAS* mutations may make their prognostic impact more challenging to determine. These two mutations may possess distinct biochemical properties and functional consequences giving rise to distinct prognostic implications. Nevertheless, the presence of both *NRAS* and *KRAS* mutations appears to exert a substantial toll on OS, as supported by various independent studies [50–53]. This emphasizes the importance of screening for *RAS* mutations both at diagnosis and during follow-up, enabling the identification of high-risk patients and the personalization of therapeutic strategies. *N/KRAS* mutations do not seem to influence responses to anthracycline-based chemotherapies, as observed in AML. Knowledge is scarcer regarding their role in responses to hypomethylating agents and combination therapies such as azacitidine (AZA) and venetoclax (Ven). The elusive nature of their prognostic relevance in this context may be due to the low frequency of *RAS*-mutated patients in princeps studies and a lack of dedicated investigations [8, 54, 55].

In CMML, *RAS* mutations are more prevalent especially in the proliferative form of the disease, at around 20–30% [56]. While *RAS* mutations appear to play a pivotal role in the transformation of CMML to AML, only *NRAS* mutations seem to exhibit a significant association with adverse clinical outcomes and are included in dedicated scores such as CPSS-Mol score, as well as the CMML transplant score. *NRAS*-mutated CMML patients encounter reduced response rates to HMA and allogeneic hematopoietic stem cell transplantation (allo-SCT), resulting in higher relapse rates and ultimately shorter OS (Table 2) [57–59]. Unlike *NRAS, KRAS* mutations are only represented in the IPSS-M score. Although conducted on a very large patient cohort, the IPSS-M score predominantly encompasses MDS but also, to a lesser extent, CMML and other MDS/MPN. While both *N/KRAS* mutations increase the risk of acute transformation in CMML, only mutant *NRAS* has been conclusively shown to influence EFS and OS in CMML patients.

In PMF, *RAS* mutations remain infrequent but are associated with higher bone marrow cellularity, increased splenomegaly, elevated circulating blast percentages, and additional driver mutations. However, in multivariate analysis, *RAS* mutations are not retained as prognostic factors for acute transformation independently of well-established markers, such as high-risk cytogenetic abnormalities and other alterations such as mutations in *ASXL1, EZH2, SRSF2*, *IDH1/2*, or *U2AF1* [21, 22, 60].

Despite their rarity, *N/KRAS* mutations in PMF are associated with reduced responses to ruxolitinib, necessitating the monitoring of *RAS* mutational status for all PMF patients. This assessment may become a routine part of monitoring to guide therapeutic decision-making. JAK2 inhibitor therapies in PMF are primarily symptom-focused and have limited impact on bone marrow fibrosis and mutation allele burden. A recent study reported that the presence of *RAS* and *CBL* mutations was linked to poorer symptom improvement and spleen size reduction, suggesting potential resistance to JAK inhibitors. This resistance may stem from two mechanisms: one study showed that a *RAS* mutation acquired within a *JAK2*^V617F^ mutated clone confers resistance to JAK inhibition, while another study highlighted PDGF-BB’s role in maintaining MEK/ERK activation in the presence of ruxolitinib (Table 2) [21, 22, 61, 62].

In adult AML, *RAS* mutations do not appear to significantly influence survival in patients subjected to intensive anthracycline-based chemotherapy, and accordingly have not been included in the European LeukemiaNet genetic risk classifications [63, 64]. However, emerging evidence suggests that *RAS* mutations may hold prognostic significance in AML patients treated with non-intensive therapies. Indeed, *RAS* mutations have been associated with higher relapse risk post-HMA treatment, such as AZA, or the Ven-AZA combination [54, 65–68]. *KRAS* but not *NRAS* mutations were also found to be associated with inferior survival in AML, particularly in the context of HMA-based therapies (Table 2) [69]. Furthermore, a recent study validated a new molecular prognostic risk signature, called mPRS, tailored for AML patients treated with HMA and Ven. This mPRS, based on the mutational status of 4 genes (*NRAS*, *KRAS*, *FLT3*, and *TP53*), can accurately segregate 3 groups of AML patients with distinct outcomes. Notably, *N/KRAS* mutations appear to negatively impact patient outcomes [70].

In JMML, a subset of *RAS*-mutated cases, combined with favorable prognostic factors; normal fetal hemoglobin levels for age and high platelet counts, have long-term survival without the need for allo-SCT. However, *NRAS* mutations in JMML are often associated with higher relapse rates, warranting adjusted post-transplant treatment strategies, including low-intensity graft versus host disease (GVHD) prophylaxis to enhance the graft versus leukemia (GVL) effect and reduce the risk of relapse. Conversely, *KRAS*-mutated JMML exhibit lower relapse rates, necessitating classical high-intensity GVHD prophylaxis (Table 2).

In pediatric AML, there is limited data regarding the potential influence of *RAS* mutations on clinical outcomes. The frequently altered tyrosine kinase and RAS/MAPK/MEK pathways, identified in 30-90% of pediatric AML patients, contributes to around 20% of relapses in this group [71, 72]. The prognostic impact of *RAS* mutations in pediatric AML has not been systematically investigated, but *RAS*-mutated pediatric AML seem to exhibit greater chemosensitivity compared to non-*RAS*-mutated AML [73, 74].

*RAS* mutations appear to negatively influence treatment responses following non-intensive therapies. In AML, RAS mutational status plays a pivotal role in the response to FLT3 inhibitors. For instance, RAS/MAPK pathway mutations emerge in approximately one third of AML patients experiencing disease progression on gilteritinib therapy [75]. A parallel study on crenolanib therapy in relapsed/refractory FLT3-mutated AML also identified epigenetic and genetic alterations, including *NRAS* mutations, associated with resistance. This resistance may be due to mutant RAS facilitating downstream ERK signaling reactivation in the presence of FLT3 inhibitors. *RAS* mutations also affect responses to venetoclax therapy by activating the Ras/Raf/MEK/ERK pathway, leading to increased MCL-1 compared to BCL2, thereby conferring resistance to BCL2 inhibitors [67, 68]. Collectively, this emphasizes the importance of early monitoring for *RAS* mutations upon initiating FLT3 inhibitor therapy, which could provide a crucial window for proactive intervention. It also suggests that targeting both MCL1 and BCL2 with venetoclax could be an alternative approach [9, 76].

In the context of IDH inhibitors such as ivosidenib and enasidenib, it is established that the existence of a *RAS* co-mutation is linked to inherent [77], but also acquired resistance [78]. Several hypotheses that might explain this resistance include the potent oncogenic signals of RAS activation diminishing 2-HG dependency and the contribution of RAS pathway-activating mutations to a sustained differentiation block following drug initiation. *RAS* mutations may also activate alternative pathways, change cellular metabolism, and induce epigenetic alterations, all of which may lead to resistance against IDH inhibitors by promoting cell survival and reducing drug sensitivity [77, 79–81].

Altogether, the impact of *RAS* mutations is far from uniform, with its significance heavily contingent upon the specific hematologic malignancy and the treatment modalities employed. Nonetheless, the presence of a *RAS* mutation correlates with an increased relapse risk in patients receiving non-intensive or targeted therapies.

## RAS targeting therapeutic strategies

Over the last decade, significant progress has been made in the development of targeted therapies in myeloid malignancies. However, molecularly targeted therapies with clinical efficacy are still lacking for RAS-mutant myeloid malignancies. The investigation of novel therapeutic strategies and combinations targeting the RAS pathway, encompassing both upstream and downstream components, is an active field of research.

MEK, the downstream effector of the RAS-MAPK pathway, has recently been the primary therapeutic focus. Trametinib, a MEK1/2 inhibitor, showed promise by inhibiting ERK phosphorylation, resulting in reduced proliferation of *NRAS*-mutated AML cells in preclinical studies [82, 83]. Other clinical trials of MEK inhibitors are currently ongoing including trametinib, cobimetinib, selumetinib, and binimetinib, in various hematologic malignancies (Table 3). A recent study also revealed that RAS pathway mutations are associated with a unique gene expression profile enriched in mitotic kinases, such as polo-like kinase 1 (PLK1). Pharmacologic inhibition of PLK1 in *RAS* mutant patient-derived xenografts yielded promising results [9]. Onvansertib, a PLK1 inhibitor, is currently undergoing phase 1 trials for relapsed/refractory RAS-mutated CMML patients (Table 3).

**Table 3:** Clinical ongoing studies for RAS-mutated myeloid malignancies.

Targeted agent	Drug class	Disease(s)	Inclusion criteria	Phase	Clinicaltrials.gov Identifier
Cobimetinib	MEK inhibitor	CMML	Newly diagnosed or HMA refractory CMML with RAS pathway activation	2	NCT04409639
Cobimetinib	MEK inhibitor	AML	Patients with R/R AML and RAS-pathway mutations	1	NCT05441514
Selumetinib	MEK inhibitor	MDS, CMML, atypical CML, and MDS/MPN-Unclassifiable, myelofibrosis	Higher risk MDS, MDS/MPNs, and myelofibrosis. Objectives include describing the preliminary clinical response rates and observing relationships between the presence of RAS activating mutations, RAS pathway activation, and clinical response.	1	NCT03326310
Onvansertib	PLK1 Inhibitor	Proliferative CMML	Patients with R/R proliferative CMML including those with a RAS mutation. Objectives include exploring the mechanism of onvansertib activity in RAS mutants.	1	NCT05549661
Trametinib	MEK inhibitor	JMML	JMML patients including those with a somatic mutation in RAS (NRAS, KRAS, RRAS or RRAS2) or PTPN11	2	NCT03190915
Trametinib	MEK inhibitor	JMML	JMML patients including those with a somatic mutation in RAS (NRAS, KRAS, RRAS or RRAS2) or PTPN11	1/2	NCT05849662

*MDS* myelodysplastic syndrome, *CMML* chronic myelomonocytic leukemia, *AML* acute myeloid leukemia, *CML* chronic myeloid leukemia, *MDS/MPN* myelodysplastic/ myeloproliferative neoplasm, *MEK* mitogen-activated protein kinase kinase, *PLK1* polo-kinase 1, *HMA* hypomethylating agent, *R/R* relapsed/refractory, *RRAS* Ras-Related gene *RRAS2* Ras-Related 2 gene, *PTPN11* tyrosine-protein phosphatase non-receptor type 11.

Novel inhibitor molecules are currently being explored in solid malignancies, offering future therapeutic promise. For example, KRAS^G12C^ inhibitors, such as sotorasib and adagrasib, have shown encouraging results in clinical trials of non-small cell lung cancer and colorectal cancer. Recent studies also investigated other inhibitory molecules such as MRTX1133 and JAB-23000 that are selective inhibitors of KRAS G12D and KRAS G12V, respectively. A phase 1 trial testing RMC-6236, a triple inhibitor of KRAS (G12V, G12D, G13C, G13D, Q61H), NRAS (Q61X) and HRAS mutants, is also underway, with optimistic outcomes [84, 85]. Nonetheless, the applicability of such molecules in the field of hematology has still been restricted due to various factors. First, given that specific NRAS inhibitors are not readily available for all mutation types, the relatively higher prevalence of *NRAS* mutations in myeloid malignancies as compared to solid tumors represents a limitation. Second, patients may harbor multiple subclones, each carrying a distinct *RAS* mutation. This genetic heterogeneity renders the therapeutic targeting of *RAS* mutations even more challenging. In this context, the use of inhibitors designed to target multiple mutations, such as RMC-6236, may be a more suitable approach. Such broad-spectrum inhibitors have the potential to address the diversity of *RAS* mutations and offer a more comprehensive strategy for tackling these genetic alterations in myeloid malignancies.

**Fig. 3:**
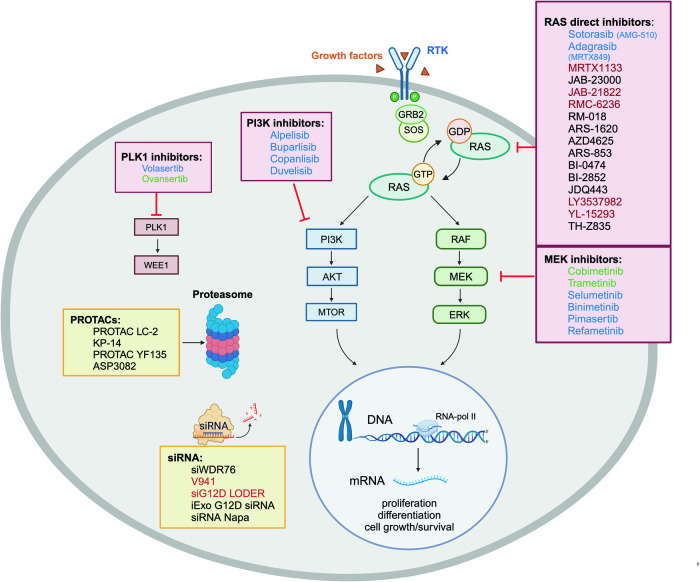
Recapitulative figure highlighting novel and ongoing therapeutic strategies for targeting RAS. The binding of growth factors to the tyrosine kinase receptor leads to its phosphorylation and the binding to the Grb2/Sos complex. RAS is controlled by a loop of an inactive, GDP-bound state and an active, GTP-bound state. Activation of RAS occurs by the binding Guanine Nucleotide Exchange Factor (GEF) proteins, including SOS, which initiate the exchange of GDP for GTP. The GTP-bound RAS activates a cascade mechanism of downstream signaling molecules including RAF and PI3K, which regulates different cellular functions such as cell proliferation, differentiation, and cell growth/survival. This figure summarizes the druggable pathways and targets in clinical trials or that are potential therapies for future use. Four critical therapeutic axes are highlighted: direct RAS inhibitors, MEK inhibitors, PI3K inhibitors and PLK1 inhibitors. Targets labeled in green are those currently in clinical trials in hematologic diseases. Targets labeled in blue are FDA-approved in the oncology field. Preclinical and clinical drugs targeting RAS-mutant in solid tumors are labeled respectively in black and red. RTK receptor tyrosine kinase, PROTACs proteolysis-targeting chimeras, siRNA small interfering RNA.

Beyond targeting the mutated RAS protein directly, alternative strategies aim to prevent its activation by inhibiting upstream signaling molecules such as SOS1. In addition, the development of PROTACs (Proteolysis-Targeting Chimeras), bi-functional molecules designed to induce proteasomal degradation of specific target proteins, are under investigation and have shown to be efficient in preclinical studies targeting RAS-mutant proteins [86–89]. RNA-based approaches, such as small interfering RNAs (siRNAs), are yet another strategy to silence the expression of mutated *RAS* at the mRNA level (Fig. 3). The siRNA inhibition strategy is a technological challenge, given the heterogeneous distribution of *K/NRAS* mutations in myeloid malignancies, and refers more towards ultra-personalized medicine than to a global management strategy [89].

Besides targeting the *RAS*-mutated clones, the development of therapies targeting the inflammatory mediators may also be beneficial to improve survival, symptoms, and quality of life for patients with RAS-mutated myeloid malignancies. This has been recently illustrated in CMML where KRAS-mutated monocytes showed constitutive activation of the NLRP3 inflammasome, increased IL-1β release, and a specific inflammatory cytokine signature. Treatment of a CMML patient with a *KRAS*^G12D^ mutation using the IL-1 receptor blocker anakinra inhibited NLRP3 inflammasome activation, reduced monocyte count, and improved patient clinical status, allowing bridging to allo-SCT [90].

Given the fact that dormancy and the pro-inflammatory microenvironment of mutant-HSCs impact the subclonal emergence of *RAS*-mutated clones, it is likely that the proliferation of a RAS-mutated clone may be contingent upon a distinct cellular state and microenvironment [48], indicating that a more effective therapeutic strategy could involve targeting both the microenvironment and the *RAS*-mutated clone. This dual approach should be considered in the future to prevent the emergence of resistance and reduce the risk of relapse. In summary, several innovative therapies and strategies are being explored either in preclinical studies or in early clinical development in solid tumors (Fig. 3). The potential for these targeted therapies to transform the treatment of myeloid malignancies remains to be investigated in combination with other molecules.

## Conclusion

This review highlights key insights on *RAS* mutations in myeloid malignancies from the past decade, encompassing their pivotal role in disease pathogenesis, prognosis, and therapy. While they may not always act as independent prognostic factors, they significantly influence clinical outcomes, disease progression, and survival. Different RAS proteins (NRAS vs. KRAS) may also differentially impact prognosis, in addition to their presence along concurrent mutations. Recent evidence indicates that *RAS* mutations also drive resistance to targeted therapies, especially FLT3, IDH1/2, or JAK2 inhibitors, as well as the venetoclax-azacitidine combination, necessitating early monitoring for intervention and exploring the clonal evolution of such subclones. While mouse models, despite limitations, offer vital platforms for studying *Ras* mutations and their interplay with other driver genetic alterations, our understanding of the intricate relationship between leukemic clones, the emergence of the RAS subclone, along the inflammatory microenvironment warrants further exploration. Advances in pharmacologic strategies have paved the way for potential therapeutic interventions targeting such mutations. Nonetheless, there is an uncharted territory regarding the application of solid tumor therapies to hematologic malignancies, promising novel trials in the future. The heterogeneity of *RAS* mutations emphasizes the need for personalized treatments and a meticulous screening of individual mutation profiles for effective therapeutic approaches.
